# The Treatment of Cancer with Mesothorium and Its Combination with Other Methods of Treatment

**Published:** 1914-01

**Authors:** 


The Treatment of Cancer With Mesothorium and Its Combination With Other Methods of Treatment.—A. Pinkuss says that the local action of the Röntgen rays, radium, or mesothorium upon cancer can destroy it to a certain depth, but one must guard against the idea that the sole treatment with the rays furnishes a sure method for the removal of cancer. The difficulty of making mesothorium and the expense of it when obtained prohibit its general use; the technic of its application is by no means fully developed and, furthermore, it is doubtful whether the mesothorium emanations can prevent the development of metastases even though the rays seem to have cured the original lesion. The combining of the emanations with the injection of thorium x and solutions of the same substance with the internal administration of pancreatin preparations, etc., can give positive cures. The old rule still holds good, however, namely, that every operable carcinoma whose risks of removal are not too great should be operated on because removal by operation at the present time is still the safest and surest way of exterminating malignant tumors.—*Ex.*

